# Outstanding Outcome of Pancreatic Cancer: What Lessons Do We Learn

**Published:** 2020-02-12

**Authors:** Muhammad W. Saif

**Affiliations:** Northwell Health Cancer Institute and Donald and Barbara Zucker School of Medicine, Hofstra/Northwell, NY, USA

The American Cancer Society’s estimates that 57,600 people (30,400 men and 27,200 women) will be diagnosed with pancreatic cancer in the United States for 2020 and approximately 47,050 people (24,640 men and 22,410 women) will die of pancreatic cancer in 2020.^1^ Despite advances in first-line therapy such as gemcitabine/nab-paclitaxel and fluorouracil, leucovorin, oxaliplatin, and irinotecan (FOLFIRINOX) in advanced pancreatic cancer (aPC), median overall survival remains less than 12-months.^2^

Prestigious Supreme Court judge announced that she is a four-time survivor of cancer. She was diagnosed with colorectal cancer in 1999 for which she underwent surgery followed by chemotherapy and radiation therapy.^3,4^ During the course, she did not miss a day on the bench, despite bring physically beaten by the cancer treatment. She overcame this weakness by working with a personal trainer, named Bryant Johnson, a former Army reservist attached to the Special Forces, who has trained Ginsburg twice weekly in the justices-only gym at the Supreme Court. A decade later on February 05, 2009 she was diagnosed with pancreatic adenocarcinoma, often deadly but in her case detected early. She underwent surgery and was released from the hospital on February 13.^5,6^ Though not known, but it was reported that spleen was removed indicating the tumor could be in the tail part of the pancreas. She went back into session on February 23, 2009. On November 8, 2018, she fractured three ribs following a fall in her office and returned to work after a day of observation.^7^ A computerized tomography (CT) scan at that time revealed suspicious lesions in her lungs for which she underwent surgical removal of these nodules on December 21, 2018.^8^ It was in August 24, 2019 when it was reported that Ginsburg had completed three weeks of focused radiation treatment to ablate a tumor found in her pancreas. Finally, on January 7, 2020, Ginsburg reported that she is again cancer free.

Pancreatic cancer remains a devastating disease but in resecting lung metastases resulting from pancreatic cancer has been reported in medical literature as well as in our experience in selected cases.^2^ Only a few cases of long-term survival after such a procedure have been reported. Certain factors are very valuable in predicting this survival including the relatively long interval between the initial resection to treat the pancreatic cancer and lung metastasis, as well as whether the metastatic lung tumor is solitary and stable over time.^9^ We consider performing lung resection in such a patient if the following conditions are met:

1. The patient is fit for surgery;
2. The primary lesion is controlled;
3. No additional metastases are found outside in other parts of the body in addition to the lung;
4. Multiple lung metastases can be removed.

Medical literature has also shown that patients in whom lung is the first site of recurrence of pancreatic cancer had better survival rates than those who developed pulmonary metastases as a second or synchronous site of recurrence. It is important to notice here that distinction between lung metastases from pancreatic cancer from primary lung cancer or a lung metastasis from a different/new cancer is prudent, especially these two tumors share few risk factors, such as smoking.^10^

Though, surgery remains the standard of care and the sole hope for cure in operable pancreatic cancer but radiation therapy can also offer local control in some patients. Particularly, in patients with local recurrence or positive margins, radiation therapy has shown considerable promise for local control. Moreover, with the improvement in radiation technology such as stereotactic body radiation therapy (SBRT), we are now able to deliver extremely precise, very intense doses of radiation to cancer cells while minimizing damage to healthy tissue in comparison to conventional radiotherapy.^11^

There are two extremely important points to be made here about her amazing outcome and they relate to her will and commitment. It is now a well-known fact that patients who exercises like Ginsburg does, tend to do better than folks who are very much affected by the tumor.^12^ In addition, family support and a will and desire to fight the cancer are additive factors. Many patients give up the fight due to depression or other factors.^13^

Another comment worth-mentioning here is the occurrence of both colorectal cancer and pancreatic cancer in an individual or in an individual with a first-degree relative deserves the testing for an identifiable genetic syndrome underlying their cancer risk, such as Lynch Syndrome.^14^ With the availability of novel diagnostic tools and risk-reducing plus therapeutic strategies, it is prudent that physicians must be vigilant about evaluating patients for hereditary cancer syndromes.
